# Fatigue of newly diagnosed patients with acute myeloid leukemia: comparison to the general population and investigation of predictive factors

**DOI:** 10.1136/bmjspcare-2020-002312

**Published:** 2023-12-07

**Authors:** Laura B. Oswald, Adriano Venditti, David Cella, Francesco Cottone, Anna Candoni, Lorella Melillo, Roberto Cairoli, Gabriella Storti, Prassede Salutari, Mario Luppi, Francesco Albano, Maria Paola Martelli, Antonio Cuneo, Agostino Tafuri, Silvia Maria Trisolini, Alessia Tieghi, Paola Fazi, Marco Vignetti, Fabio Efficace

**Affiliations:** 1 Health Outcomes and Behavior Program, Moffitt Cancer Center, Tampa, FL, USA; 2 Hematology, Department of Biomedicine and Prevention, University Tor Vergata, Rome, Italy; 3 Policlinico Tor Vergata, Rome, Italy; 4 Department of Medical Social Sciences, Northwestern University Feinberg School of Medicine, Chicago, IL, USA; 5 Data Centre and Health Outcomes Research Unit, Italian Group for Adult Haematological Diseases (GIMEMA), Rome, Italy; 6 Hematology, Azienda Sanitaria Universitaria Integrata di Udine, Udine, Italy.; 7 Fondazione IRCCS Casa Sollievo della Sofferenza, UO di Ematologia, San Giovanni Rotondo (FG), Italy; 8 Ospedale Niguarda Ca Granda, Milan, Italy.; 9 Azienda Ospedaliera S. G. Moscati, Avellino, Italy.; 10 Azienda USL di Pescara, Pescara, Italy.; 11 Ematologia, Dipartimento di Scienze Mediche e Chirurgiche Materno-Infantili e dell’Adulto, Università degli Studi di Modena e Reggio Emilia, Modena, Italy; 12 Ematologia, Dipartimento dell’Emergenza e dei Trapianti di Organi, Università degli Studi di Bari Aldo Moro, Bari, Italy.; 13 Hematology and Clinical Immunology, Department of Medicine and Surgery, University of Perugia, Perugia, Italy.; 14 Azienda Ospedaliero Universitaria Arcispedale Sant’Anna, Ferrara, Italy; 15 Azienda Ospedaliera Sant’ Andrea, Rome, Italy.; 16 Hematology, Department of Translational and Precision Medicine, Sapienza University, Rome, Italy.; 17 Azienda Unità Sanitaria Locale – IRCCS di Reggio Emilia, Reggio Emilia, Italy.

**Keywords:** Quality of life, Fatigue, Acute Myeloid Leukemia, Supportive care, Symptom management

## Abstract

**Objectives:**

This study compared the burden of fatigue between treatment-naïve patients with newly diagnosed acute myeloid leukemia (AML) and the general population and investigated patient factors associated with fatigue severity.

**Methods:**

Pretreatment patient-reported fatigue was assessed with the FACIT-Fatigue questionnaire in a sample of 463 newly diagnosed patients with AML who were enrolled in a clinical trial. Multivariable linear regression models were used to estimate the adjusted mean differences in fatigue between patients with AML and adults from the general population (N=847) by AML disease-risk categories. A clinically meaningful difference in fatigue was defined as ≥3 points. Univariable and multivariable linear regression models were used to identify sociodemographic, clinical, and molecular correlates of worse fatigue in patients with AML.

**Results:**

Patients with AML reported adjusted mean fatigue scores that were 7.5 points worse than the general population (95% CI −8.6 to −6.4, p<0.001). Across AML disease-risk categories, adjusted mean differences in fatigue compared to the general population ranged from 6.7 points worse (patients with favorable risk: 95% CI −8.6 to −4.8, p<0.001) to 8.9 points worse (patients with poor risk, 95% CI −10.5 to −7.2, p<0.001). Overall, 91% of patients with AML reported fatigue that was equal to or worse than the general population’s median fatigue score. Higher pretreatment fatigue was independently associated with female sex, WHO performance status ≥1, and lower platelet levels.

**Conclusions:**

Patients with newly diagnosed AML reported worse fatigue than the general population, and mean differences exceeded twice the threshold for clinical significance. Our findings may help to identify patients with AML most likely to benefit from supportive care interventions to reduce fatigue.

## Introduction

Acute myeloid leukemia (AML) is a heterogeneous cancer that starts in the bone marrow and is characterized by the clonal expansion of myeloid blasts in the blood, bone marrow, and/or other tissues [1]. AML is the one of most common leukemias affecting adults in the United States (US), accounting for 32% of leukemia incidence and second only to chronic lymphocytic leukemia (CLL, 37%) [2]. This translates to almost 20,000 expected new cases of AML in the US in 2020 alone. AML is also a leading cause of leukemia-related deaths, with more than 11,000 AML-related deaths expected in 2020 [2]. The National Comprehensive Cancer Network (NCCN) has defined risk categories to describe the prognostic patterns of AML (i.e., *favorable*, *intermediate*, and *poor* risk categories) [1]. NCCN risk categories are based on molecular mutations and cytogenetic abnormalities (e.g., chromosomal deletions, translocations, and duplications), and better outcomes are expected for patients who fall in the *favorable* risk category compared to others. Clinicians use the current NCCN risk categories to inform risk-adapted treatment plans for individual patients with AML. Thus, NCCN risk categories are important for maximizing clinical outcomes.

A recent review concluded that fatigue is the most common symptom reported by patients with AML, regardless of treatment status (i.e., active treatment vs. post-treatment) [3]. Studies of patients with AML undergoing intensive and prolonged treatments, such as induction chemotherapy, found that fatigue is more strongly related to health-related quality of life (HRQOL) than other treatment side effects (e.g., nausea, emesis, appetite loss) [4]. In addition, patients with AML consistently rate fatigue as moderate to severe throughout treatment [5]. Post-treatment, long-term and severe fatigue is a significant concern affecting HRQOL for many patients [3, 6]. Moreover, a recent study showed that greater patient-reported fatigue in treatment-naïve patients with AML predicted shorter survival [7]. Therefore, understanding and alleviating fatigue early in the trajectory of AML diagnosis and treatment may be a critical component to promoting favorable patient outcomes.

Given fatigue’s prevalence and persistence, researchers have attempted to identify factors that predict fatigue in patients with AML, including sociodemographic characteristics, clinical characteristics [8], and biological factors (e.g., pro-inflammatory cytokines) [9–12]. However, past studies have not distinguished patients with AML by NCCN risk categories, possibly due to small sample sizes limiting the statistical power to do so. Further characterization of fatigue among subgroups of patients with AML using common clinical categorizations will provide important information about variations in the burden of fatigue in this patient population. These nuances may be particularly important in light of research showing that patient-reported fatigue in patients with untreated AML is associated with overall survival [7]. Moreover, though fatigue is generally accepted as a hallmark of AML and its treatments, studies have not yet determined the severity of fatigue among patients with newly diagnosed AML relative to the general population.

To address these limitations, the primary objective of this analysis was to compare the burden of fatigue between a cohort of treatment-naïve patients with newly diagnosed AML and a cohort representing the general population. Secondary objectives were to investigate fatigue severity by NCCN risk categories and to investigate sociodemographic, clinical, and molecular correlates of worse pretreatment fatigue among patients with AML.

## Methods

Between January 2012 and May 2015, 515 treatment-naïve patients with AML were registered to participate in a single-arm study to determine whether a risk-adapted, minimal-residual-disease directed therapy could positively affect overall survival [13]. For the purpose of these analyses, we considered 508 participants who were eligible and had an available risk category. Patient-reported HRQOL was a secondary endpoint of the trial, and all patients were invited to complete a baseline assessment (i.e., before starting therapy) with the European Organization and Research and Treatment of Cancer (EORTC) Quality of Life Questionnaire-Core 30 (EORTC QLQ-C30) [14] and the Functional Assessment of Chronic Illness Therapy (FACIT)-Fatigue scale [15, 16]. The trial was originally designed in 2010, and thus all patients were stratified according to contemporary classifications (i.e., NCCN 2009 version 1) [17]. Eligibility criteria for participants were an unequivocal diagnosis of untreated, de novo AML according to WHO diagnostic criteria [18], age 18 to 60 years old, no prior treatments for AML, and a WHO performance status of 0–3 [19]. Additional details related to the study inclusion and exclusion criteria are reported elsewhere [13].

All participants provided written informed consent. The study was approved by the ethics committees of each participating center and conducted in accordance with the Declaration of Helsinki.

### Sample of Adults from the General Population

Data for the general population were collected by a marketing information and decision support system titled Knowledge Networks (Menlo Park, CA). Knowledge Networks drew a random sample of individuals at least 18 years old in the US from an internet-based survey panel including more than 100,000 demographically representative adults. Participants on the panel responded to one survey per month in exchange for free installation of WebTV internet service. In total, 1,075 individuals from the general population completed the FACIT-Fatigue questionnaire. Of those individuals, 61 were excluded from these analyses because they reported a current or historic cancer diagnosis, and 167 were excluded because they were older than 61 years old (i.e., the maximum age in the AML sample). Thus, 847 participants (49.6% male, mean of 39.8 years old) were retained for these analyses. These participants are a largely overlapping subset of participants that have been used as a general population reference group to compare other patient populations’ fatigue (e.g., patients with myelodysplastic syndromes [20] and anemic cancer patients [21]).

### Measures

#### Fatigue.

Self-reported fatigue was assessed using the 13-item FACIT-Fatigue scale [15, 16]. Participants rated statements about fatigue in the past week on a Likert-type scale from *not at all* (0) to *very much* (4). Consistent with other FACIT measures, negatively worded items were reverse scored and responses were summed so that higher total scores indicated better functioning (possible range 0–52). Thus, lower total scores indicated worse fatigue [15]. Consistent with past work, a difference of at least 3 points was considered clinically meaningful [15, 16]. For these analyses, only the baseline fatigue scores of patients with AML were compared to fatigue in the general population, so that scores were not confounded by factors such as active AML treatments.

### Statistical Analyses

Frequencies, proportions, means, standard deviations, medians, and interquartile ranges (IQR) were used to describe the main characteristics of patients with AML. Chi-square and Wilcoxon-Mann-Whitney tests were used to assess possible systematic differences between patients with AML who did vs. did not complete the FACIT-Fatigue questionnaire at baseline. For these analyses, patients with intermediate risk were merged with patients with intermediate risk and no leukemia-associated immunophenotypes (previously identified in the main clinical paper [13]). Multivariable linear regression analyses were performed to estimate the overall mean difference in fatigue scores between patients with AML and the general population, adjusting for age and sex and including a binary status indicator (AML vs. general population). The multivariable linear regression model was repeated separately by patients’ NCCN risk category (i.e., *favorable*, *intermediate*, and *poor* risk). For descriptive purposes, the cumulative distribution of FACIT-Fatigue scores was computed for patients with AML and the general population, and the proportions of patients with AML who reported a fatigue score equal to or worse than the mean and median fatigue scores in the general population were reported. To determine independent correlates of fatigue among patients with AML, univariable linear regression analyses were conducted with sociodemographic, clinical, and molecular variables as independent variables. The following sociodemographic and clinical variables were considered: sex (male vs. female), age, WHO performance status (0 vs. ≥1), presence of comorbidities (yes vs. no), hemoglobin levels, white blood cell count, percentage of blast cells, and platelet count. The following molecular variables were considered: presence (yes vs. no) of FLT3-ITD, RUNX1-RUNX1T1, CBFβ-MYH11, and NMP1 gene mutations. Then, a multivariable linear regression model was estimated to explain fatigue among patients with AML including the significant sociodemographic, clinical, and molecular predictors of fatigue identified from the univariable analyses. All statistical tests were two-sided, and statistical significance was determined with α=0.05. Analyses were performed with SAS software v.4 (SAS Institute Inc., Cary NC, USA).

## Results

Of the 508 patients with AML considered for these analyses, 463 (91%) completed the baseline FACIT-Fatigue questionnaire. There were no differences between patients who did (n=463) vs. did not complete the questionnaire (n=45) with regard to key sociodemographic and clinical characteristics. Details are reported in the supplemental materials.

Table 1 describes the characteristics of patients with AML overall and by NCCN risk categories. There were roughly equivalent proportions of males (n=264, 52.0%) and females (n=244, 48.0%), and patients were a median of 49 years old (IQR 40.0–55.0). Most patients had a WHO performance status of 0 (n=289, 58.6%) and no comorbidities (n=402, 84.1%).

### Fatigue in Patients with AML Compared to the General Population

The average fatigue score in the general population was 40.5 (*SD*=10.2, median 44.0), and the average fatigue score among patients with AML was 33.1 (*SD*=8.7, median 33.8) (Table 2). After adjusting for age and sex, fatigue among patients with AML across NCCN risk categories was an average of 7.5 points worse than the general population (95% CI −8.6, −6.4, *p*<0.001), which is more than twice the 3-point threshold for a clinically meaningful difference. The largest difference in fatigue scores was observed between the general population and patients in the NCCN *poor* risk category; fatigue among patients with AML and *poor* risk was an average of 8.9 points lower than the general population (95% CI −10.5 to −7.2, *p*<0.001), almost three times the threshold for a clinically meaningful difference.

**Figure 1.**
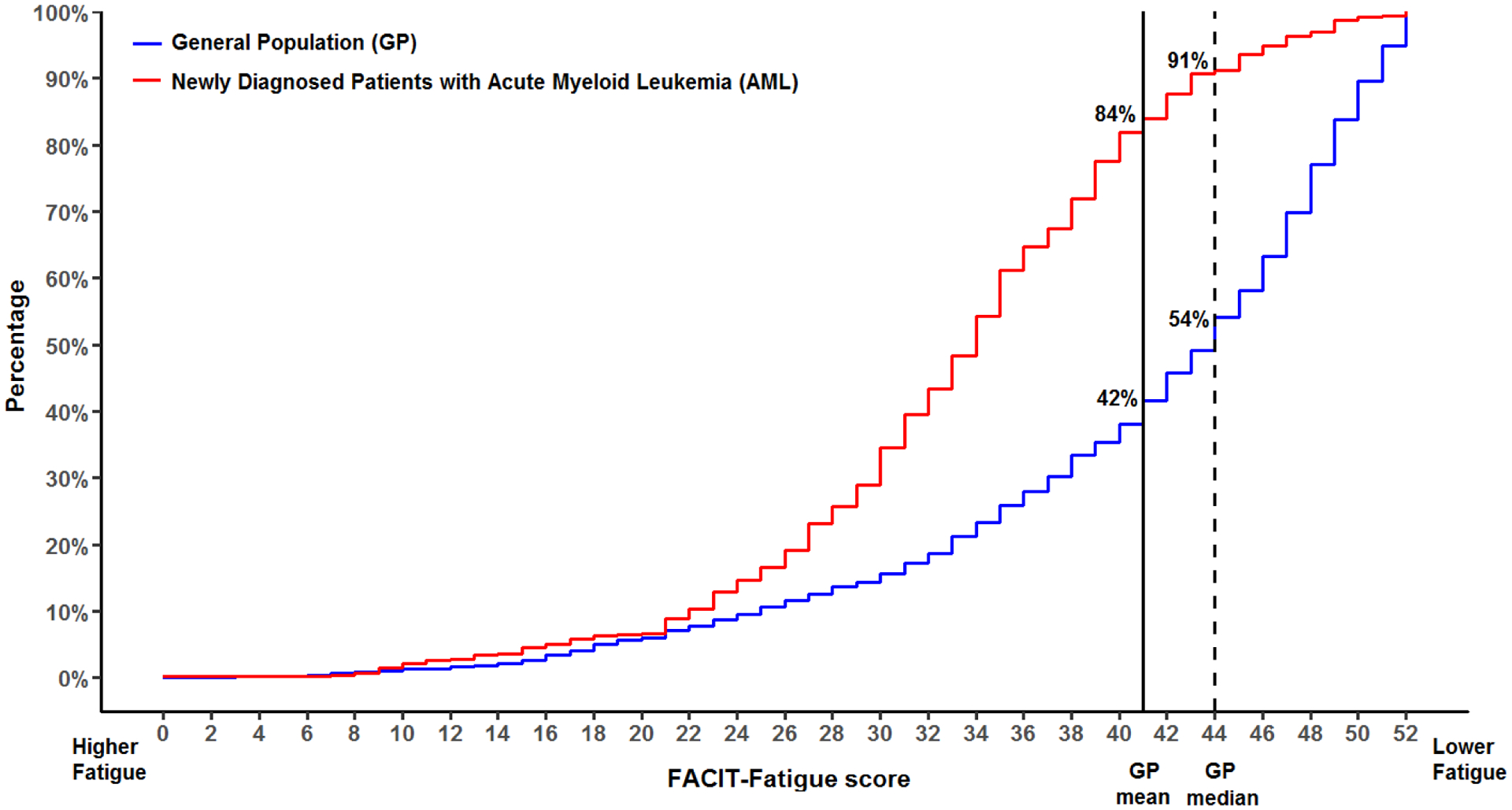
Cumulative distribution of FACIT-Fatigue scores in patients with acute myeloid leukemia (AML) and the general population. From left to right, the height of the curve represents the overall proportion of patients reporting fatigue equal to or worse than the corresponding FACIT-Fatigue score. The vertical line represents the mean FACIT-Fatigue score in the general population.

**Figure 2.**
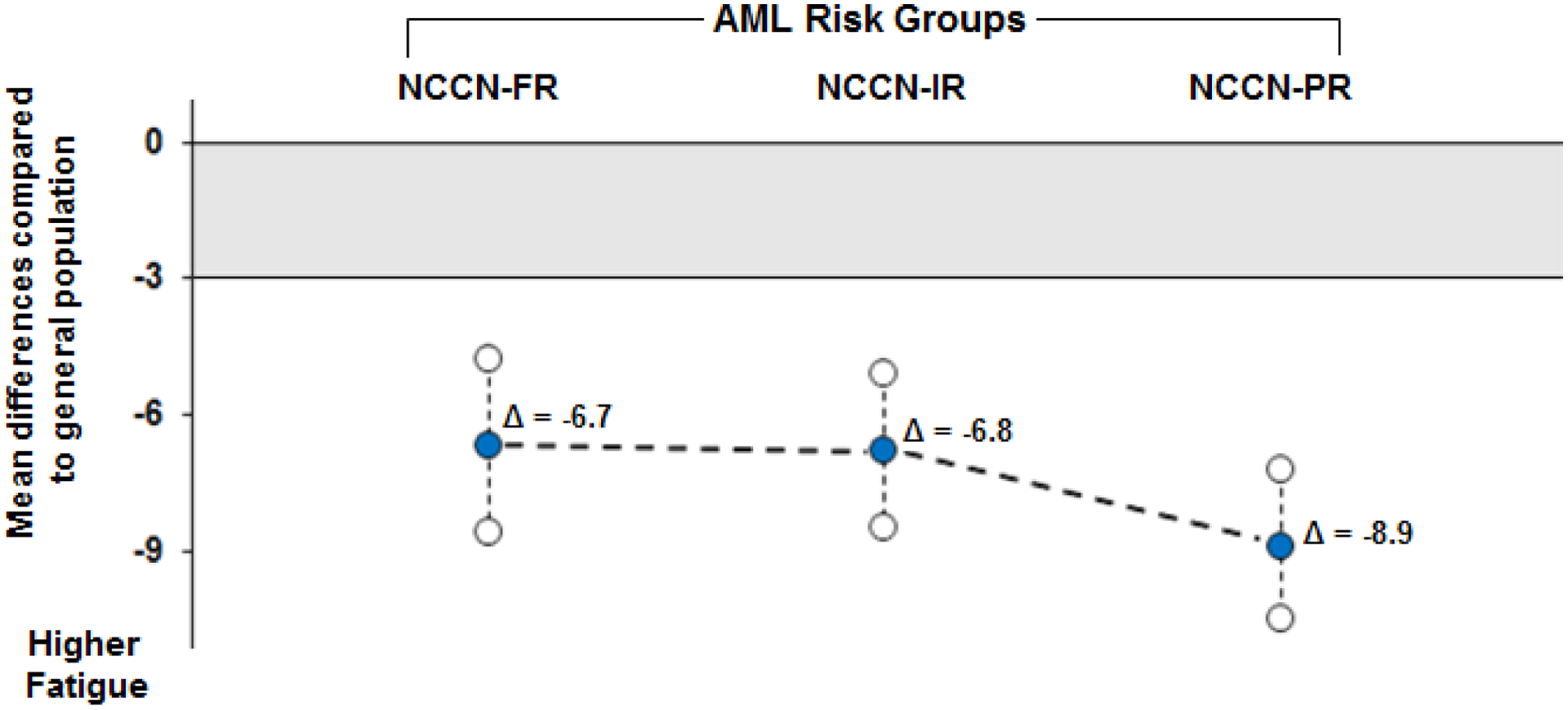
Mean differences in FACIT-Fatigue scores between patients with acute myeloid leukemia (AML) and the general population by National Comprehensive Cancer Network (NCCN) risk category (FR, favorable risk; IR, intermediate risk; PR, poor risk). Mean differences were adjusted for age and sex. Lines between the adjusted mean differences were plotted for descriptive purposes. Mean differences plotted outside the grey shaded area indicate that the mean difference exceeded the 3-point clinically meaningful difference threshold.

The cumulative distribution of fatigue scores in patients with AML and the general population are shown in Figure 1. Overall, 84% (n=389/463) and 91% (n=422/463) of patients with AML reported fatigue that was equal to or worse than the mean and median fatigue scores in the general population, respectively. For descriptive purposes, the mean differences between patients with AML by NCCN risk category and the general population are graphically depicted in Figure 2.

### Independent Correlates of Fatigue in Patients with AML

Worse fatigue among patients with AML was associated with female sex (β=−2.11, 95% CI 3.70 to −0.53, *p*=0.009), WHO performance status of at least 1 (β=−3.15, 95% CI −4.79 to −1.52, *p*<0.001), lower hemoglobin levels (β=0.56, 95% CI 0.12 to 0.99, *p*=0.013), greater percentage of blast cells (β=−0.05, 95% CI −0.08 to −0.02, *p*<0.001), higher white blood cell counts (β=−0.35, 95% CI −0.51 to −0.19, *p*<0.001), and lower platelet counts (β=0.18, 95% CI 0.08 to 0.28, *p*=0.001). The only significant molecular correlate was the FLT3-ITD mutation (β=−2.08, 95% CI −3.90 to −0.26, *p*=0.025). All univariable linear regression models are described in Table 3.

In multivariable analysis, the following variables were significantly associated with worse fatigue above and beyond other predictors: female sex (β=−2.30, 95% CI −4.01 to −0.59, *p*=0.009), WHO performance status of at least 1 (β=−2.55, 95% CI −4.30 to −0.81, *p*=0.004), and lower platelet count (β=0.22, 95% CI 0.08 to 0.37, *p*=0.002). Additional information about the multivariable analysis is shown in Table 3.

## Discussion

This was the first study to compare fatigue between newly diagnosed, treatment-naïve patients with AML to the general population, overall and by disease risk categories. Findings indicated that most patients with AML had worse fatigue than the general population, and differences in fatigue were clinically meaningful across NCCN risk categories. Analyses by individual risk categories showed that the burden of fatigue in both *favorable* and *intermediate* risk categories was nearly identical and exceeded twice the magnitude of a clinically meaningful difference with the general population. Patients in the NCCN *poor* risk category reported worse fatigue than the general population at a magnitude of almost three times the clinically meaningful threshold. The sizeable differences in fatigue observed across the three risk categories highlights how vulnerable patients with AML are to fatigue, even before initiating treatment.

Prior work has established that fatigue is a highly prevalent symptom among patients with AML [3] and persists well beyond diagnosis [22]. Studies suggest that fatigue improves for patients who achieve remission with treatment [23], and fatigue improvement appears to be unrelated to treatment intensity (i.e., intensive vs. non-intensive chemotherapy) [24]. Moreover, fatigue severity appears to be similar among younger (<60 years old) and older patients with AML (≥60 years old) [25]. Recently, Buckley and colleagues [26] interviewed 82 patients with AML at various disease stages to determine the most prevalent symptoms associated with AML. Fatigue was one of the most prevalent symptoms, second only to fear/anxiety, and fatigue was reported by more than three quarters of the sample (76%). Moreover, almost half of the sample (44%) reported that fatigue was “very impactful” for HRQOL.

While the prevalence and trajectory of fatigue in AML have been fairly well studied, far fewer studies have identified independent factors that could help clinicians identify patients at risk for worse fatigue burden early in the diagnostic workup. The current study addresses this gap in the literature by focusing on fatigue among patients with AML before receiving any treatments. In addition, patients in this sample were younger than those reported in other work (median age was 49 years old). In a multivariable analysis, worse fatigue was correlated with female sex, WHO performance status of at least 1, and lower platelet counts. These characteristics could aid clinicians in identifying subgroups of patients at greater risk of worse fatigue as well as worse outcomes that are associated with fatigue, such as shorter survival [7]. The finding that female sex was associated with worse fatigue is consistent with previous studies of patients with other hematologic malignancies, either newly diagnosed [27] or undergoing treatment [28–30], and should be explored more in future work. In addition, the finding that lower platelet counts was associated with worse fatigue is consistent with a recent study by Zhang and colleagues [31], who showed that platelets count at the time of diagnosis was an independent prognostic factor for survival in patients with AML, thereby lending important insights about the relevance of this variable for capturing overall disease burden.

This was the first study to evaluate molecular mutations as correlates of fatigue in AML. However, it is not the first study to investigate biological correlates more generally. There is a small body of work evaluating the relationships between fatigue and cytokines in patients with AML [7, 9–11], which is driven by the sickness behavior model. This model posits that behavioral symptoms of illness, such as fatigue, may be determined by sickness-associated inflammation and cytokine activity [32]. However, a recent study of more than 200 patients with AML concluded that cytokines explained only a small amount of the variance in cancer-related fatigue [12]. In recent years, advances in the use of genomic sequencing have allowed researchers and clinicians to determine how specific molecular mutations relate to outcomes among patients with AML [33]. Yet until now, no studies have investigated how molecular mutations relate to fatigue in this population. Our univariable analyses showed that the presence of the FLT3-ITD mutation was associated with worse fatigue, though this relationship did not persist in multivariable models. The FLT3-ITD mutation is one of the most common AML-related mutations, observed in almost one-third of patients with AML [33]. Past work indicates that patients with the FLT3-ITD mutation are at risk for worse treatment outcomes including greater risk of disease relapse and mortality [34]. Consistent with the sickness behavior model, it is possible that the relationship between worse fatigue and FLT3-ITD mutation observed in this study reflects greater overall disease burden. This possibility and other clinical implications of the FLT3-ITD mutation should be explored in future work to inform better management of fatigue and to promote better clinical outcomes. In addition, given that sickness behaviors typically present in clusters (e.g., fatigue, pain, depression) [35], future work should also consider the role of other patient-reported outcomes and symptom clusters.

Limitations of this study include a cross-sectional design, and thus the direction of causality could not be determined in the reported relationships. In addition, data related to other common symptoms that have been previously associated with fatigue in cancer patients, such as pain and depression, was not available [35]. Large-scale prospective studies are needed to better elucidate factors associated with fatigue among patients with AML. Finally, while the NCCN classification system used in this study was current at the time of study design, this classification system has since been refined. However, this did not affect the primary objective of this work.

This study has notable strengths. To the best of our knowledge, this was the largest study of fatigue among newly diagnosed and treatment-naïve patients with AML. It used a well-validated patient-reported measure of fatigue, which allowed for evaluations of both the statistical and clinical significance of the reported differences in fatigue between patients with AML and the general population. In addition, the majority of patients had no significant medical comorbidities that could account for the reported burden of fatigue, strengthening the clinical implications of these results. Finally, as previously noted, this was the first study to compare pretreatment fatigue in newly diagnosed patients with AML to the general population and assess the burden of fatigue in AML by NCCN risk categories. It was also the first to evaluate molecular mutations as correlates of fatigue in this population. In conclusion, our findings represent a step toward better understanding the burden of fatigue among newly diagnosed patients with AML.

## Supplementary Material

Supplemental Table

## Figures and Tables

**Table 1.** Characteristics of all patients with AML and by NCCN risk category.

Variable		NCCN Risk Category		
	Total (N=508)	Favorable (n=139)	Intermediate (n=175)	Poor (n=194)
Sex, n (%)				
Male	264 (52.0)	72 (51.8)	92 (52.6)	100 (51.5)
Female	244 (48.0)	67 (48.2)	83 (47.4)	94 (48.5)
Age, years				
Median	49.0	50.0	48.0	48.0
IQR	40.0–55.0	43.0–55.0	40.0–55.0	38.0–55.0
WHO Performance Status, n (%)				
0	289 (58.6)	80 (59.3)	100 (59.2)	109 (57.7)
1	143 (29.0)	43 (31.8)	50 (29.6)	50 (26.5)
2	59 (12.0)	11 (8.2)	19 (11.2)	29 (15.3)
3	2 (0.4)	1 (0.7)	0 (0)	1 (0.5)
Missing	15 (-)	4 (-)	6 (-)	5 (-)
Presence of comorbidities, n (%)				
No comorbidities	402 (84.1)	106 (80.9)	136 (82.9)	160 (87.4)
At least 1 comorbidity	76 (15.9)	25 (19.1)	28 (17.1)	23 (12.6)
Missing	30 (-)	8 (-)	11 (-)	11 (-)
Hb level, g/dL				
Median	8.9	8.9	9.2	8.6
IQR	8.1–10.0	8.2–10.0	8.2–10.5	8.0–9.8
WBC count, cells x10^9^/L				
Median	14	17.4	5.7	27.3
IQR	3.4–50.0	5.6–57.0	2.0–22.5	6.1–70.9
Blast cells, %				
Median	54.0	54.5	40.0	62.0
IQR	20.0–80.0	20.0–74.0	10.0–77.0	39.0–90.0
Platelet count, x10^3^/L				
Median	55.0	56.0	55.0	50.0
IQR	29.0–94.0	28.0–94.0	30.0–108.0	28.0–82.0

*Abbreviations*. NCCN, National Comprehensive Cancer Network; IQR, interquartile range; WHO, World Health Organization; Hb, hemoglobin; WBC, white blood cell.

**Table 2.** Distribution of fatigue scores and adjusted mean differences between patients with AML and the general population (n=463).

	Distribution of fatigue scores			Adjusted mean difference from general population* (95% CI)	*p*
Sample	Mean (SD)	Median	Range		
General population	40.5 (10.2)	44.0	2–52	NA	NA
AML total sample	33.1 (8.7)	33.8	0–52	−7.5 (−8.6, −6.4)	<0.001
NCCN risk category					
Favorable	33.9 (7.8)	34.7	13–52	−6.7 (−8.6, −4.8)	<0.001
Intermediate	34.0 (8.3)	34.3	8–51	−6.8 (−8.5, −5.1)	<0.001
Poor	31.8 (9.4)	31.9	0–50	−8.9 (−10.5, −7.2)	<0.001

* Mean differences were adjusted for age and sex;

*Abbreviations*: SD, standard deviation; CI, confidence interval; NA, not applicable. AML, acute myeloid leukemia; NCCN, National Comprehensive Cancer Network.

**Table 3.** Results of univariable and multivariable regression models relating sociodemographic, clinical, and molecular variables to fatigue among patients with AML.

	Univariable Analysis			Multivariable Analysis		
	Estimate	95% CI	*p*	Estimate	95% CI	*p*
**Sociodemographic variables**						
Female sex	−2.11	3.70, −0.53	0.009	−2.30	−4.01, −0.59	0.009
Age	−0.03	−0.11, 0.05	0.437	NA	NA	NA
**Clinical variables**						
WHO performance status ≥1	−3.15	−4.79, −1.52	<0.001	−2.55	−4.30, −0.81	0.004
≥1 comorbidity	−1.26	−3.47, 0.95	0.262	NA	NA	NA
Hb level, g/dL	0.56	0.12, 0.99	0.013	0.06	−0.43, 0.54	0.815
WBC count, x10^9^/L	−0.35	−0.51, −0.19	<0.001	−0.15	−0.34, 0.04	0.125
Blast cells, %	−0.05	−0.08, −0.02	<0.001	−0.02	−0.05, 0.02	0.314
Platelet count, x10^9^/L	0.18	0.08, 0.28	0.001	0.22	0.08, 0.37	0.002
**Molecular variables**						
FLT3-ITD	−2.08	−3.90, −0.26	0.025	−0.05	−2.05, 1.95	0.961
RUNX1-RUNX1T1	0.20	−3.27, 3.68	0.908	NA	NA	NA
CBFβ-MYH11	−0.14	−3.21, 2.93	0.928	NA	NA	NA
NMP1	−0.17	−1.83, 1.49	0.839	NA	NA	NA

Only significant univariable predictors of fatigue were included in the multivariable analysis.

*Abbreviations*: WHO, World Health Organization; Hb, hemoglobin; WBC, white blood cell.
