# Supplementary material for: Fatigue of newly diagnosed patients with acute myeloid leukemia: comparison to the general population and investigation of predictive factors
Source: BMJ Support Palliat Care. Author manuscript; Available in PMC 2022 Nov 3. (PMC8563490; doi:10.1136/bmjspcare-2020-002312)
Supplement: Supplemental Table [file NIHMS1720287-supplement-Supplemental_Table.docx]

**Supplemental Table.** Comparison of patients with AML who did and did not complete the FACIT-Fatigue questionnaire pretreatment.

| Variable | | Patients Who Did Not Complete the FACIT-Fatigue Questionnaire  (N=45) | Patients Who Completed the FACIT-Fatigue Questionnaire  (N=463) | *p*-value |
| --- | --- | --- | --- | --- |
| Sex, n (%) | |  |  | 0.665 |
|  | Male | 22 (48.9) | 242 (52.3) |  |
|  | Female | 23 (51.1) | 221 (47.7) |  |
| Age, years | |  |  | 0.255 |
|  | Median | 51.0 | 48.0 |  |
|  | IQR | 39.0-56.0 | 40.0-55.0 |  |
| WHO Performance Status, n (%) | |  |  | 0.400 |
|  | 0 | 24 (54.6) | 265 (59.0) |  |
|  | 1 | 17 (38.6) | 126 (28.0) |  |
|  | 2 | 3 (6.8) | 56 (12.5) |  |
|  | 3 | 0 (0.) | 2 (0.5) |  |
|  | Missing | 1 (-) | 14 (.) |  |
| Presence of comorbidities, n (%) | |  |  | 0.459 |
|  | No comorbidities | 37 (88.1) | 365 (83.7) |  |
|  | At least 1 comorbidity | 5 (11.9) | 71 (16.3) |  |
|  | Missing | 3 (-) | 27 (-) |  |
| Hb level, g/dL | |  |  | 0.923 |
|  | Median | 8.9 | 8.9 |  |
|  | IQR | 8.3-10.0 | 8.0-10.0 |  |
| WBC count, cells x 10^9^/L | |  |  | 0.182 |
|  | Median | 25.5 | 13.0 |  |
|  | IQR | 5.4-48.3 | 3.4-50.9 |  |
| Blast cells, % | |  |  | 0.772 |
|  | Median | 55.0 | 54.0 |  |
|  | IQR | 18.0-80.0 | 20.0-80.0 |  |
| Platelet count, x10^3^/L | |  |  | 0.069 |
|  | Median | 66.0 | 54.0 |  |
|  | IQR | 40.0-109.0 | 28.0-87.0 |  |

*Abbreviations.* AML, acute myeloid leukemia; dL, deciliter; g, grams; Hb, hemoglobin; IQR, interquartile range; n, frequency; L, liter; NCCN, National Comprehensive Cancer Network; WBC, white blood cell; WHO, World Health Organization.
